# Crosstalk between Mitochondrial Ca^2+^ Uptake and Autophagy in Skeletal Muscle

**DOI:** 10.1155/2019/1845321

**Published:** 2019-09-08

**Authors:** Gaia Gherardi, Giulia Di Marco, Rosario Rizzuto, Cristina Mammucari

**Affiliations:** Department of Biomedical Sciences, University of Padova, 35131 Padova, Italy

## Abstract

Autophagy is responsible for the maintenance of skeletal muscle homeostasis, thanks to the removal of aberrant and dysfunctional macromolecules and organelles. During fasting, increased autophagy ensures the maintenance of the amino acid pool required for energy production. The activity of the mitochondrial Ca^2+^ uniporter (MCU), the highly selective channel responsible for mitochondrial Ca^2+^ uptake, controls skeletal muscle size, force, and nutrient utilization. Thus, both autophagy and mitochondrial Ca^2+^ accumulation play a pivotal role to maintain muscle homeostasis and to sustain muscle function. Here, we address whether, in skeletal muscle, mitochondrial Ca^2+^ uptake and autophagy are mutually related. Muscle-restricted MCU silencing partially inhibits the autophagy flux. Moreover, skeletal muscle-specific deletion of the essential autophagy gene Atg7, known to cause the accumulation of dysfunctional mitochondria, drastically reduces mitochondrial Ca^2+^ accumulation. Thus, a vicious cycle takes place, in which reduced MCU activity hampers the autophagic flux, and loss of autophagy further impairs mitochondrial Ca^2+^ signaling.

## 1. Introduction

Macroautophagy, hereafter referred to as autophagy, is a well-established mechanism responsible of the bulk degradation of intracellular macromolecules and organelles [1]. During starvation, autophagy contributes to the supplying of cellular nutrients, in particular amino acids, required for cell survival. In addition, basal autophagy, i.e., the constitutive autophagic degradation process that occurs in physiological conditions, is responsible for the degradation of dysfunctional and damaged proteins and organelles, thus ensuring the maintenance of tissue homeostasis [2].

Skeletal muscle accounts for about 40% of the total body mass, representing the main body protein pool. Muscle autophagy is induced by fasting [3, 4], and in catabolic conditions, skeletal muscle-derived amino acids are utilized as energy source by different organs [5]. In addition, as postmitotic tissue, maintenance of skeletal muscle homeostasis requires the continuous removal of aberrant macromolecules and organelles even in fed conditions. Accordingly, deletion of Atg7, an essential autophagy gene [6], causes muscle atrophy and accumulation of protein aggregates and aberrant mitochondria [7]. In particular, mitochondria of skeletal muscle-specific ATG7^−/−^ mice are characterized by defective respiration, increased reactive oxygen species production [8], and altered membrane potential [9, 10]. The latter observation is particularly relevant in light of the fact that autophagy is modulated in response to the mitochondrial energy status [11–13]. Moreover, mitochondria are elongated in response to fasting, thus increasing the activity of the ATP synthase, ensuring physiological levels of ATP production [14].

Ca^2+^-dependent processes occur both in the cytosol and within mitochondria. In particular, mitochondrial Ca^2+^ uptake regulates a number of cell functions, ranging from the buffering of cytosolic Ca^2+^ increases, thus indirectly regulating cytosolic Ca^2+^-dependent processes, to intraorganelle processes, including the control of cell death [15]. In physiological conditions, mitochondrial Ca^2+^ uptake positively regulates three key dehydrogenases of the TCA cycle eventually leading to increased ATP production, thus playing a pivotal role in the control of the cellular energy status [16].

The mitochondrial Ca^2+^ uniporter (MCU), the highly selective Ca^2+^ channel located in the inner mitochondrial membrane (IMM), is responsible for mitochondrial Ca^2+^ uptake [17, 18]. In skeletal muscle, MCU overexpression positively regulates muscle size and protects from denervation-induced loss of muscle mass [19]. This is associated with increased activity of well-known hypertrophic signaling routes, like the Akt pathway, previously reported to play an inhibitory role on autophagy [3] and to the reduced activity of the protein synthesis inhibitor and autophagy activator, GSK3 *β* [20, 21]. In parallel, the cell volume occupied by mitochondria is increased [19]. On the other hand, reduced or absent MCU expression, specifically in skeletal muscle, decreases muscle size, impairs muscle function, determines a slow to fast switch in MHC expression, and as a consequence of decreased PDH activity, triggers a metabolic rewiring towards preferential fatty acid utilization to counteract the reduced glucose oxidation [19, 22]. In addition, MCU silencing determines the accumulation of altered mitochondria and an overall reduction of the volume occupied by these organelles [19]. The metabolic rewiring occurring in MCU-depleted skeletal muscle eventually translates into a systemic catabolic response [22].

Ca^2+^-dependent regulation of autophagy has been observed in a wide variety of conditions; however, whether cytosolic Ca^2+^ plays a permissive or rather an inhibitory role on autophagy induction is still debated and possibly depends on the cell type and context [23]. Moreover, increased cytosolic Ca^2+^ levels, in response to ER Ca^2+^-depleting stimuli, induce mitochondrial Ca^2+^ accumulation that partially contributes to autophagy modulation. In particular, Cárdenas et al. reported that constitutively low levels of ER-mitochondria Ca^2+^ transfer are essential for autophagy suppression. In detail, cells lacking all three inositol 1,4,5-triphosphate receptor (InsP3R) isoforms are characterized by increased autophagy levels, that are required for cell survival during nutrient deprivation, and pharmacological inhibition of the mitochondrial Ca^2+^ uniporter phenocopies InsP3R inhibition [24]. In addition, in a genetic model of mitochondrial disorder due to a point mutation in the ND5 complex I subunit, a decrease in mitochondrial Ca^2+^ uptake is associated with an increase in the catabolic response and in the induction of prosurvival autophagy. Moreover, in patient fibroblasts, MCU overexpression restores normal levels of autophagy [25]. However, the negative modulation of mitochondrial Ca^2+^ uptake and of mitochondrial membrane potential by protein kinase C beta (PKC*β*) activation leads to autophagy inhibition, as opposite to starvation [26].

Thus, while the role of mitochondrial metabolism in the modulation of autophagy is undoubted, whether mitochondrial Ca^2+^ uptake increases or reduces autophagy depends, at least partially, on the cell context. Despite the significant work made to uncover the crosstalk between autophagy and mitochondrial Ca^2+^ accumulation, little is known on the role of mitochondrial Ca^2+^ uptake in the regulation of skeletal muscle autophagy. In addition, whether autophagy affects mitochondrial Ca^2+^ uptake in skeletal muscle is still obscure.

Here, we demonstrate that skeletal muscle autophagy is unaffected by MCU overexpression, while it is partially impaired by decreased MCU expression. In addition, mitochondrial Ca^2+^ uptake is significantly reduced in autophagy-deficient skeletal muscles, thus contributing to muscle dysfunction.

## 2. Materials and Methods

### 2.1. Animals

All animal experiments were approved and performed in accordance with the Italian law D. L.vo n_26/2014. CD1 mice were purchased by Charles River Laboratories. Skeletal muscle-specific MCU knockout (skMCU^−/−^) mice and their relative controls (MCU^fl/fl^) were previously described [22]. Skeletal muscle-specific Atg7 knockout (skATG7^−/−^) mice and their relative controls (ATG7^fl/fl^) were previously described [7].

### 2.2. AAV Infection

AAV9-MCU and AAV9-shMCU were purchased from Vector Biolabs (Malvern, PA) and were previously described [19]. EDL muscles of adult CD1 mice were isolated through a small hindlimb incision, and 10^10^ vg were injected along the muscle length. Muscles were analyzed 14 days postinjection.

### 2.3. Autophagic Flux Measurements

Colchicine treatment: autophagic flux analyses using colchicine treatment were performed as previously described [27] with some modifications. Adult EDL muscles were injected with AAV9-MCU or AAV9-shMCU. 14 days postinfection, 0.1 mg/kg colchicine (Sigma-Aldrich) was i.p injected. Control mice received an equal volume of 0.9% NaCl. The same treatment was repeated 12 hours later. Mice were sacrificed 24 hours after the first injection; EDL muscles were harvested and frozen in liquid nitrogen-cooled isopentane. In the case of the starving mouse group, food was withdrawn at the same time of the first colchicine injection.

Leupeptin treatment: autophagic flux analyses using leupeptin treatment were performed as previously described [28] with some modifications. Adult EDL muscles were injected with AAV9-MCU or AAV9-shMCU. 14 days postinfection, 30 mg/kg leupeptin (Sigma-Aldrich) was i.p injected. Control mice received an equal volume of 0.9% NaCl. Mice were sacrificed 5 hours after the injection; EDL muscles were harvested and frozen in liquid nitrogen-cooled isopentane. In the case of the starving mouse group, food was withdrawn at the same time of the leupeptin injection.

### 2.4. *In Vivo* DNA Transfection of Mouse Skeletal Muscle

Hyaluronidase solution (2 mg/ml) (Sigma-Aldrich) was injected under the hindlimb footpads of anesthetized mice. After 30 minutes, 20 *μ*g of plasmid DNA in 20 *μ*l of physiological solution was similarly injected. Then, one gold-plated acupuncture needle was placed under the skin at heel, and a second one, at the base of the toes, oriented parallel to each other and perpendicular to the longitudinal axis of the foot and connected to the BTX porator (Harvard Apparatus). The muscles were electroporated by applying 20 pulses, 20 ms each, 1 s of interval to yield an electric field of 100 V. Single fiber cultures were carried out 7 days later.

### 2.5. Real-Time Imaging of Mitochondrial Ca^2+^ in FDB Fibers

FDB fibers were isolated 7 days after *in vivo* transfection with a plasmid encoding 4mtGCaMP6f [22]. Muscles were digested in collagenase A (4 mg/ml) (Roche) dissolved in Tyrode's salt solution (pH 7.4) (Sigma-Aldrich) containing 10% fetal bovine serum (Thermo Fisher Scientific). Single fibers were isolated, plated on laminin-coated glass coverslips, and cultured in DMEM with HEPES (42430 Thermo Fisher Scientific), supplemented with 10% fetal bovine serum, containing penicillin (100 U/ml) and streptomycin (100 *μ*g/ml). Fibers were maintained in culture at 37°C with 5% CO_2_.

After single fiber isolation, real-time imaging was performed. During the experiments, myofibers were maintained in Krebs-Ringer modified buffer (135 mM NaCl, 5 mM KCl, 1 mM MgCl_2_, 20 mM HEPES, 1 mM MgSO_4_, 0.4 mM KH_2_PO_4_, 1 mM CaCl_2_, 5.5 mM glucose, and pH 7.4) at RT, in the presence of 75 *μ*M N-benzyl-P-toluenesulfonamide (BTS, Tocris) to avoid the fiber contraction. 20 mM caffeine (Sigma-Aldrich) was added when indicated to elicit Ca^2+^ release from intracellular stores. Experiments were performed on a Zeiss Axiovert 200 microscope equipped with a 40×/1.3 N.A. Plan Fluor objective. Excitation was performed with a DeltaRAM V high-speed monochromator (Photon Technology International) equipped with a 75 W xenon arc lamp. Images were captured with a high-sensitivity Evolve 512 Delta EMCCD (Photometrics). The system is controlled by MetaMorph 7.5 (Molecular Devices) and was assembled by Crisel Instruments. 4mtGCaMP6f was alternatively excited every second at 475 and 410 nm, respectively, and images were acquired through an emission filter (535/30 nm) (Chroma). Exposure time was set to 50 ms. Acquisition was performed at binning 1 with 200 of EM gain. Image analysis was performed with Fiji distribution of the ImageJ software [29]. Images were background corrected frame by frame by subtracting the mean pixel value of a cell-free region of interest. Changes in Ca^2+^ levels (475/410 nm fluorescence ratio) were expressed as *R*/*R*_0_, where *R* is the ratio at time *t* and *R*_0_ is the ratio at the beginning of the experiment. Mitochondrial Ca^2+^ peak was expressed as (*R*‐*R*_0_)/*R*_0_ and normalized for the control value.

### 2.6. Western Blotting and Antibodies

To monitor protein levels, frozen muscles were pulverized by means of Qiagen TissueLyser and protein extracts were prepared in an appropriate buffer containing 50 mM Tris pH 7.5, 150 mM NaCl, 5 mM MgCl_2_, 1 mM DTT, 10% glycerol, 2% SDS, 1% Triton X-100, Complete EDTA-free protease inhibitor mixture (Roche), 1 mM PMSF, 1 mM NaVO_3_, 5 mM NaF, and 3 mM *β*-glycerophosphate. 40 *μ*g of total proteins was loaded, according to BCA quantification. Proteins were separated by SDS-PAGE electrophoresis, in commercial 4-12% acrylamide gels (Thermo Fisher Scientific), and transferred onto nitrocellulose membranes (Thermo Fisher Scientific) by semidry electrophoretic transfer. Blots were blocked for 1 hour at RT with 5% nonfat dry milk (Bio-Rad) in TBS-tween (0.5 M Tris, 1.5 M NaCl, and 0.01% Tween) solution and incubated at 4°C with primary antibodies. Secondary antibodies were incubated 1 hr at RT. The following primary antibodies were used: anti-LC3 (1 : 1000, Cell Signaling), anti-p62 (1 : 5000, Sigma-Aldrich), anti-actin (1 : 20000, Santa Cruz), and anti-MCU (1 : 1000 Sigma-Aldrich). Secondary HRP-conjugated antibodies were purchased from Bio-Rad and used at 1 : 5000 dilution.

### 2.7. RNA Extraction, Reverse Transcription, and Quantitative Real-Time PCR

Total RNA was extracted from tibialis anterior (TA) muscles using the SV Total RNA Isolation Kit (Promega) following the manufacturer's instructions. The RNA was quantified with NanoDrop (Thermo Fisher Scientific). Complementary DNA was generated from 500 nmol of total RNA with a cDNA Synthesis Kit SuperScript II (Thermo Fisher Scientific). Oligo(dT)12–18 primers (Thermo Fisher Scientific) were used as primer for first-strand cDNA synthesis with reverse transcriptase. The obtained cDNA was analyzed by real-time PCR using the IQ5 thermocycler and the SYBR green chemistry (Bio-Rad). The primers were designed and analyzed with Primer3 [30]. The housekeeping gene Gapdh was used as an internal control for cDNA normalization. For quantification, expression levels were calculated by the *Δ*Ct method. Real-time PCR primer sequences were as follows: Lc3: Fw 5′-CACTGCTCTGTCTTGTGTAGGTTG-3′, Rv 5′-TCGTTGTGCCTTTATTAGTGCATC-3′; Gapdh: Fw 5′-CACCATCTTCCAGGAGCGAG-3′, Rv 5′-CCTTCTCCATGGTGGTGAAGAC-3′; and p62: Fw 5′-CCCAGTGTCTTGGCATTCTT-3′, Rv 5′-AGGGAAAGCAGAGGAAGCTC-3′.

### 2.8. Statistical Analysis of Data

Statistical data are presented as mean ± SD. Significance was calculated by Student's *t*-test or the Mann-Whitney rank sum test.

## 3. Results

### 3.1. MCU Silencing Negatively Affects Autophagy Flux in Skeletal Muscle

Muscle autophagy is induced during starvation [3], a condition characterized by a high rate of catabolism [31]. Previous work highlighted the link between mitochondrial Ca^2+^ accumulation and optimal cellular bioenergetics maintenance, demonstrating that reduced ER-mitochondrial Ca^2+^ transfer increases prosurvival autophagy in conditions of nutrient deprivation [24]. We have previously demonstrated that diminished mitochondrial Ca^2+^ uptake in skeletal muscle inhibits anabolic pathways and triggers atrophy [19]. In addition, muscle-specific MCU deletion impairs substrate oxidation and mitochondrial respiration [22]. These data suggest that, in skeletal muscle, MCU silencing could trigger autophagy induction. However, in MCU-silenced mice, the number and size of mitochondria are reduced and the frequency of severely damaged organelles is increased [19]. Since basal autophagy contributes to the removal of dysfunctional organelles and proteins [7, 32], one alternative possibility would be that MCU silencing actually negatively affects skeletal muscle autophagy.

To discern among these alternatives, we directly measured autophagy in MCU-silenced EDL muscles. We took advantage of the adenoassociated virus- (AAV-) based transduction of shMCU to specifically reduce mitochondrial Ca^2+^ uptake in the skeletal muscle in vivo, as previously reported [19]. We monitored the lipidation of LC3 and the protein levels of p62, two well-known autophagy markers. The lipidated form of LC3, LC3-II, is embedded in the autophagosome membrane, and its levels are proportional to the number of autophagosome. p62 delivers polyubiquitinated cargoes to autophagy and accumulates in autophagy-deficient cells [33].

**Figure 1:**
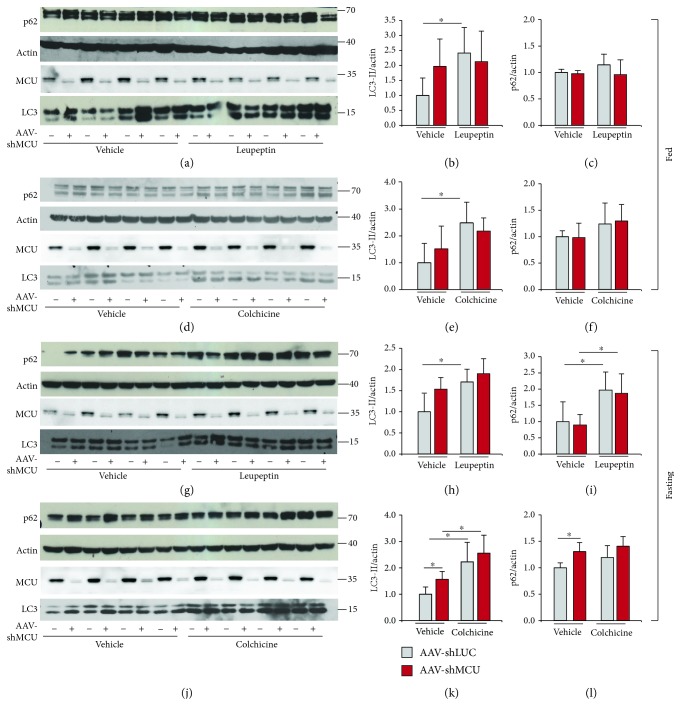
Autophagy flux analyses in MCU-silenced muscles. (a-f) Immunoblotting analysis of EDL muscles infected with AAV-shMCU or AAV-shLUC as control and treated or not with leupeptin (a-c) or colchicine (d-f) in fed conditions. Western blot analyses demonstrated efficient MCU downregulation in EDL muscles. Protein levels of LC3-II and p62 were used to monitor autophagy, relative to actin protein levels used as loading control. (b, c, e, and f) Quantification by densitometry of the ratio between LC3-II/actin and p62/actin. ^∗^*p* < 0.05, *t* test (two-tailed, unpaired) of four animals per condition. Data are presented as mean ± SD. (g-l) Immunoblotting analysis of EDL muscles infected with AAV-shMCU or AAV-shLUC as control and treated or not with leupeptin (g-i) or colchicine (j-l) upon starvation. Western blot analyses demonstrated efficient MCU downregulation in EDL muscles. (h, i, k, and l) Quantification by densitometry of the ratio between LC3-II/actin and p62/actin. ^∗^*p* < 0.05, *t* test (two-tailed, unpaired) of four animals per condition. Data are presented as mean ± SD.

In MCU-silenced muscles of fed mice, LC3-II levels tend to increase compared to control muscles infected with AAV-LacZ (Figures 1(a), 1(b), 1(d), and 1(e)). This could be due either to increased autophagosome production or to increased accumulation of autophagosomes because of impaired fusion with lysosomes and/or degradation of autophagolysosomes. To distinguish between these two possibilities, we performed an analysis of the autophagic flux, i.e., the balance between formation and degradation of autophagosomes. Accordingly, we compared autophagy in muscles treated or not with either colchicine or leupeptin that causes the accumulation of autophagosomes. In detail, colchicine is a microtubule-depolymerizing agent that blocks the fusion of autophagosomes with lysosomes; instead, leupeptin is a lysosomal protease inhibitor. Thus, the LC3-II levels in leupeptin and colchicine-treated muscles represent the maximal accumulation of nondigested autophagosomes [33].

**Figure 2:**
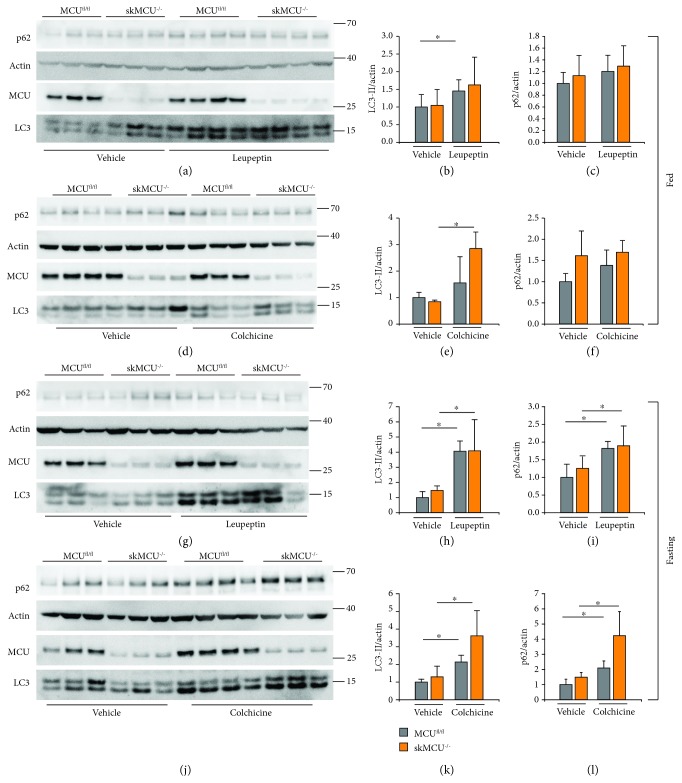
Autophagy flux analyses in skMCU^−/−^ mice. (a-f) Immunoblot of EDL muscles of skMCU^−/−^ or skMCU^fl/fl^ mice treated or not with leupeptin (a-c) or colchicine (d-f) in fed conditions. Western blot analyses demonstrated efficient MCU deletion in EDL muscles. LC3-II and p62 protein levels were used to monitor autophagy, relative to actin protein levels used as loading control. (b, c, e, and f) Quantification by densitometry of the ratio between LC3-II/actin and p62/actin. ^∗^*p* < 0.05, *t* test (two-tailed, unpaired) of three vehicle animals and four treated animals, respectively. Data are presented as mean ± SD. (g-l) Immunoblotting analysis of EDL muscles of skMCU^−/−^ or skMCU^fl/fl^ mice treated or not with leupeptin (g-i) or colchicine (j-l) upon starvation. Western blot analyses demonstrated efficient MCU deletion in EDL muscles. (h, i, k, and l) Quantification by densitometry of the ratio between LC3-II/actin and p62/actin. ^∗^*p* < 0.05, *t* test (two-tailed, unpaired) of, respectively, three vehicle animals and four treated animals. Data are presented as mean ± SD.

In the AAV-shLUC-infected muscles in fed condition, we observed an increase in LC3-II protein levels in colchicine- or leupeptin-treated muscles, due to the block of autophagy, as expected. AAV-shMCU-treated muscles reached maximal LC3-II levels even in the absence of inhibitor treatment (Figures 1(a), 1(b), 1(d), and 1(e)). Thus, MCU silencing triggers a block in the autophagy flux which may account for the increased number of damaged mitochondria in MCU-silenced muscles [19]. As a second marker of autophagy flux, we monitor the p62 protein levels, which accumulate in response to defective autophagy [34]. Besides autophagy-dependent degradation, p62 protein levels depend on multiple factors, including gene transcription and proteasome activity. In addition, previous observations indicate that accumulation of p62 occurs in a shifted timeframe compared to LC3-II production [33]. In our experiments, autophagy flux inhibition did not affect p62 levels in fed conditions (Figures 1(a), 1(c), 1(d), and 1(f); 2(a), 2(c), 2(d), and 2(f); and 3(a) and 3(c)) indicating that p62 does not represent here an optimal autophagic marker.

Next, we evaluated the effects of MCU silencing on the autophagic flux in conditions of nutrient deprivation and thus of energy stress. For this purpose, we measured autophagy flux in muscles of fasting animals. Fasting and treatment with autophagy inhibitors were coincident, i.e., 5 hours for leupeptin-treated muscles and 24 hours for colchicine-treated muscles. Both 5 hours and 24 hours of starvation increased LC3-II and p62 protein levels ([Supplementary-material supplementary-material-1]). MCU silencing caused a tendency towards increased LC3-II protein levels in muscles of mice deprived of food for 5 hours (Figures 1(g) and 1(h)), which became significant when starvation was prolonged to 24 hours (Figures 1(j) and 1(k)). When fasting mice infected with AAV-shMCU in skeletal muscle were treated with leupeptin, we observed no difference in LC3-II protein levels compared to untreated shMCU-injected muscles, indicating that MCU silencing in muscles of mice fasted for 5 hours triggers a block of autophagy (Figures 1(g) and 1(h)). p62 protein levels were increased in both AAV-shLUC- and AAV-shMCU-infected muscles upon 5 hours of fasting and treatment with leupeptin (Figures 1(g)–1(i)). These data suggest that increased autophagosome production by fasting, associated with short-term treatment with an autophagy flux inhibitor, is the ideal setting to uncover p62 accumulation in skeletal muscle, at least in our experimental conditions. However, p62 protein levels were unaffected by MCU silencing (Figures 1(g)–1(i)). When mice were fasted for 24 hours and treated with colchicine, MCU-silenced muscles were autophagy-competent (Figures 1(j) and 1(k)), suggesting that prolonged starvation removes the block in autophagy caused by the reduction in mitochondrial Ca^2+^ uptake. Moreover, in muscles of mice treated with colchicine and starved for 24 hours, p62 protein levels were unaffected, again indicating the complexity of p62 homeostasis (Figures 1(j)–1(l)).

### 3.2. Autophagy Flux Is Functional in Skeletal Muscle-Specific MCU KO

The previous experiments demonstrate that MCU silencing partially inhibits the autophagic flux in skeletal muscle. We further investigated the modulation of autophagy in the skeletal muscle-specific MCU KO mouse (hereafter referred to as skMCU^−/−^) in which a completed ablation of mitochondrial Ca^2+^ accumulation occurs [22]. Reduced respiration and a metabolic rewiring that comprises a catabolic systemic response characterize this mouse model.

We performed autophagy flux measurements in skMCU^−/−^ muscles both in basal and in starving conditions. In muscles of fed mice, LC3-II levels were similar in controls and in skMCU^−/−^ muscles (Figures 2(a), 2(b), 2(d), and 2(e)). When muscles were treated with the autophagy inhibitors, LC3-II levels tend to increase both in MCU^fl/fl^ and in skMCU^−/−^ muscles. However, as reported above (Figure 1), p62 levels were not affected by treatment with colchicine or leupeptin (Figures 2(a), 2(c), 2(d), and 2(f)). Then, we measured autophagy flux in muscles of fasting animals. LC3-II protein levels were similar in skMCU^−/−^ muscles compared to controls. In addition, LC3-II protein levels were increased in MCU^fl/fl^ muscles treated with leupeptin or colchicine, as expected, as well as in skMCU^−/−^ muscles (Figures 2(g), 2(h), 2(j), and 2(k)). p62 protein levels were increased by fasting associated with either leupeptin or colchicine treatment, both in MCU^fl/fl^ and in skMCU^−/−^ muscles (Figures 2(g)–2(i) and 2(j)–2(l)). To determine whether LC3-II and p62 transcription was regulated, we measured the relative mRNA levels of these two transcripts in skMCU^−/−^ muscles treated with the autophagy flux inhibitors. As reported in [Supplementary-material supplementary-material-1], leupeptin or colchicine treatment did not increase LC3 or p62 expression, either in fed or in starved conditions, indicating that the increases in protein levels are not due to a transcriptional activation.

These data indicate that autophagy flux is maintained despite the constitutive depletion of mitochondrial Ca^2+^ uptake.

### 3.3. MCU Overexpression Does Not Alter Autophagic Flux in Skeletal Muscle

Increased mitochondrial Ca^2+^ uptake triggers muscle hypertrophy and protein synthesis [19]. Enhanced energy production and activation of anabolic signaling routes should in principle play an inhibitory effect on autophagy. On the other hand, PDH activity is unaffected by MCU overexpression [19], indicating that increased mitochondrial Ca^2+^ uptake does not impinge on the oxidative metabolism of muscle mitochondria.

**Figure 3:**
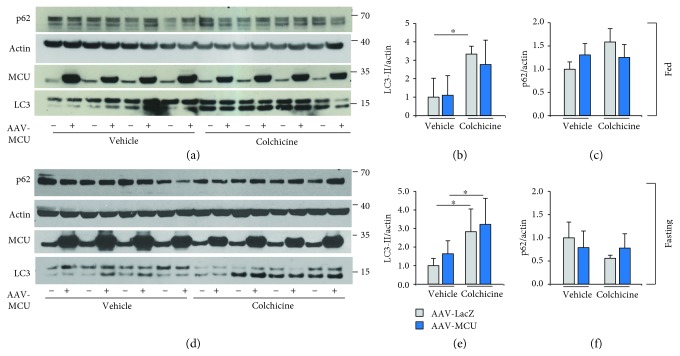
Autophagy flux analyses in MCU overexpressing muscles. (a-c) Immunoblotting analysis of EDL muscles infected with AAV-MCU or AAV-LacZ as control and treated or not with colchicine in fed conditions. Western blot analyses demonstrated efficient MCU overexpression in EDL muscles. Protein levels of LC3-II and p62 were used to monitor autophagy, relative to actin protein levels used as loading control. (b, c) Quantification by densitometry of the ratio between LC3-II/actin and p62/actin. ^∗^*p* < 0.05, *t* test (two-tailed, unpaired) of, respectively, three vehicle animals and four treated animals. Data are presented as mean ± SD. (d-f) Immunoblotting analysis of EDL muscles infected with AAV-MCU or AAV-LacZ as control and treated or not with colchicine upon starvation. Western blot analyses demonstrated efficient MCU overexpression in EDL muscles. (e, f) Quantification by densitometry of the ratio between LC3-II/actin and p62/actin. ^∗^*p* < 0.05, *t* test (two-tailed, unpaired) of four animals per condition. Data are presented as mean ± SD.

We wondered whether the overexpression of MCU in skeletal muscle could modify either basal or induced autophagy flux. We performed autophagic flux analyses in AAV-MCU-injected muscles in fed and starving conditions. In fed animals, no difference was observed in LC3-II protein levels in MCU overexpressing muscles compared to AAV-LacZ-infected control muscles (Figures 3(a) and 3(b)), and an increase in LC3 II protein levels was observed both in mock infected and in MCU overexpressing muscles treated with colchicine (Figures 3(a) and 3(b)). In addition, p62 protein levels were unaffected by MCU overexpression (Figures 3(a) and 3(c)). These data demonstrate that MCU overexpressing muscles are autophagy-competent, not differently from control muscles, and that an increase in mitochondrial Ca^2+^ uptake does not alter the autophagy flux in fed conditions. Next, we measured whether MCU overexpression impinges on the autophagy process during starvation. We detected no difference in the LC3-II protein levels in starved muscles upon AAV-MCU infection compared to AAV-LacZ-infected muscles. In addition, we observed an increase in LC3-II protein levels in the muscles treated with colchicine, both infected with AAV-LacZ and infected with AAV-MCU (Figures 3(d) and 3(e)). Similarly to fed muscles, p62 was unaffected by MCU overexpression in fasting conditions (Figures 3(d) and 3(f)). Taken together, these results show that MCU overexpression does not affect autophagy in skeletal muscle.

### 3.4. Lack of Skeletal Muscle Autophagy Causes a Decrease in Mitochondrial Ca^2+^ Uptake

**Figure 4:**
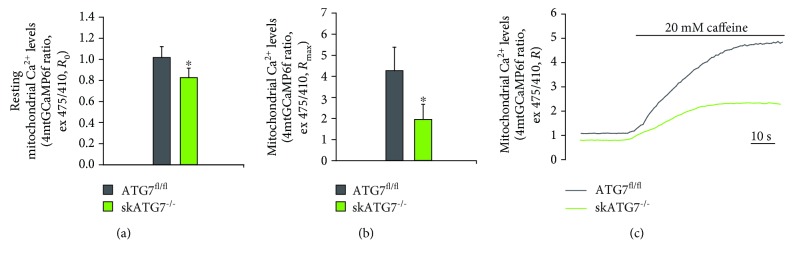
Mitochondrial Ca^2+^ uptake measurements in skATG7^−/−^ mice. (a) Resting mitochondrial [Ca^2+^] was decreased in skATG7^−/−^ FDB myofibers compared to ATG7^fl/fl^ controls. Data are presented as mean ± SD (>10 fibers per condition). (b) Ratiometric measurements of mitochondrial Ca^2+^ uptake upon caffeine treatment highlighted a reduction in peak mitochondrial [Ca^2+^] of skATG7^−/−^ FDB myofibers compared to ATG7^fl/fl^ controls. ^∗^*p* < 0.05, *t* test (two-tailed, unpaired) of >10 fibers per condition. Data are presented as mean ± SD. (c) Representative traces of mitochondrial Ca^2+^ uptake measurements.

It is clearly established that the deletion of the autophagy genes Atg7 or Atg5 in different tissues, including skeletal muscle, liver, and heart, results in swollen and aberrant mitochondria [6–8, 35]. In particular, autophagy-deficient muscles are atrophic and accumulate protein aggregates and dysfunctional mitochondria [7, 8], characterized by a decreased oxygen consumption rate and increased levels of reactive oxygen species [8]. We wondered whether these defects are associated with impaired mitochondrial Ca^2+^ signaling. For this purpose, we measured mitochondrial Ca^2+^ uptake in mice where Atg7, an essential gene required for autophagosome formation, is deleted specifically in skeletal muscle (hereafter referred as skATG7^−/−^) [7]. We transfected in vivo the adult flexor digitorum brevis (FDB) muscles of skATG7^−/−^ mice with plasmids encoding a GFP-based Ca^2+^ probe targeted to mitochondria, mtGCaMP6m [22]. One week later, we performed real-time imaging experiments on isolated single myofibers. At the beginning of the experiment, we measured basal mitochondrial [Ca^2+^] and then we added caffeine to discharge the sarcoplasmic reticulum Ca^2+^ pool, driving an increase in cytosolic [Ca^2+^] and a following accumulation of Ca^2+^ into mitochondria. skATG7^−/−^ skeletal muscle fibers showed both a decrease in mitochondrial Ca^2+^ uptake and a diminished resting mitochondrial [Ca^2+^] compared to ATG7^fl/fl^ myofibers (Figures 4(a)–4(c)). These data suggest that loss of autophagy is associated with decreased mitochondrial Ca^2+^ uptake, either as a causal factor or as a consequence of dysfunctional mitochondria activity.

## 4. Discussion

A lot of effort has been made to understand how mitochondrial Ca^2+^ accumulation could regulate skeletal muscle homeostasis. MCU^−/−^ mice, despite an overall mild phenotype, have significant impairment in exercise capacity and in muscle force [36]. Modulation of MCU specifically in skeletal muscle controls myofiber size impinging on anabolic signaling pathways [19, 22]. Moreover, despite decreased glucose oxidation and oxygen consumption rate [22], skeletal muscle-specific MCU^−/−^ mice switch their muscle metabolism towards increased dependency on fatty acid oxidation [37] and present a systemic catabolic response which involves both liver and adipose tissue metabolic remodeling.

Mitochondrial Ca^2+^ signaling regulates autophagy; however, whether it plays a permissive or an inhibitory role depends on the different settings [11, 23]. In cells deleted of all three InsP3R isoforms, autophagy is induced as a consequence of impaired ER-mitochondria Ca^2+^ transfer that, by reducing ATP production, causes the activation of AMPK [24]. However, in a different context, PKC*β* overexpression diminishes mitochondrial Ca^2+^ uptake which eventually inhibits autophagy [26]. Finally, in fibroblasts of patients expressing a mutated subunit of the ETC complex I, mitochondrial Ca^2+^ uptake decrease is accompanied by an increase in the autophagy flux [25]. With these premises, the consequence of MCU modulation in skeletal muscle autophagy is uncertain.

Our data indicate that modulation of skeletal muscle autophagy relies on changes in mitochondrial Ca^2+^ signaling. In particular, we demonstrated that decreased MCU expression in adult animals, and thus impaired oxidative metabolism and energy production, partially hampers autophagy flux, as already suggested by Patergnani et al. [26]. However, different from PKC*β* overexpressing cells, characterized by reduced mitochondrial membrane potential, this parameter is unaffected in MCU-silenced muscles [19], indicating that the mechanism underlying the inhibition of autophagy flux by MCU silencing in skeletal muscle is still unknown. In detail, in AAV-shMCU-treated muscles, autophagy flux was blocked both in fed and in fasting conditions, with one exception. When fasting was prolonged to 24 hours, in MCU-silenced muscles, autophagy flux was restored. One possibility is that prosurvival autophagy induced by starvation overcomes the inhibitory effect of MCU silencing. Importantly, the block in autophagy flux could contribute to the increased number of aberrant mitochondria observed in AAV-shMCU muscles [19]. On the other hand, in skMCU^−/−^ muscles, autophagy flux is unaffected. This could be because long-term skeletal muscle-specific MCU deletion triggers increased muscle glucose uptake and systemic catabolic responses involving liver and adipose tissue that could in part mitigate the negative effects of reduced mitochondrial Ca^2+^ signaling on autophagy activity.

Next, we demonstrated that MCU overexpression has no effect on the autophagy flux, either in basal conditions or during starvation. One should take into account the fact that increased mitochondrial Ca^2+^ uptake plays different roles according to the cell context. For example, in the liver, mitochondrial Ca^2+^ overload triggered by MICU1 knockdown is associated with the opening of the permeability transition pore (PTP), a large-conductance channel, and eventually cell death [38]. Vice versa, in skeletal muscle, MCU overexpression does not cause any sign of myofiber damage or death [19], suggesting that healthy skeletal muscle mitochondria are prone to sustain elevated Ca^2+^ waves, without impinging on degradation processes like autophagy. Whether dysregulation of mitochondrial Ca^2+^ signaling affects mitophagy, i.e., the selective autophagic degradation of mitochondria, is an open issue and deserves further investigation.

In the second part of this work, we addressed whether impaired autophagy could alter mitochondrial Ca^2+^ uptake. We detected a decreased mitochondrial basal [Ca^2+^] and a diminished mitochondrial Ca^2+^ uptake in skeletal muscles deleted of the essential autophagy gene Atg7 [7]. This is a part of a complex scenario, in which defective mitochondria, both in terms of morphology and in terms of function, accumulate in autophagy-deficient cells and tissues [6–8, 35, 39]. This would suggest that altered mitochondrial Ca^2+^ signaling is a late consequence of the increased aberrant mitochondrial population. However, both skATG7^−/−^ and skMCU^−/−^ muscles are atrophic and have reduced oxygen consumption rates. Thus, it is plausible that reduced mitochondrial Ca^2+^ uptake in skATG7^−/−^ muscles precedes signs of mitochondrial damage and dysfunction, actively contributing to the acceleration of the degenerative process.

## 5. Conclusions

Skeletal muscle is characterized by great plasticity, and autophagy contributes to muscle homeostasis and response to stress stimuli. Mitochondrial Ca^2+^ signaling controls muscle trophism, activity, and metabolic adaptations. Autophagy flux is impaired by reduced mitochondrial Ca^2+^ uptake in adult skeletal muscle. Vice versa, mitochondrial Ca^2+^ signaling is decreased in autophagy-deficient myofibers, where dysfunctional organelles accumulate. However, whether this is just a consequence of the decrease number of healthy mitochondria or rather it plays a causative role is still to be determined. Possibly, a vicious cycle occurs, in which reduced mitochondrial Ca^2+^ uptake impairs the autophagy flux, and this causes the accumulation of dysfunctional mitochondria that, in turn, reduce mitochondrial Ca^2+^ signaling.

## Data Availability

The data used to support the findings of this study are included within the article.

## Supplementary Materials

Supplementary Materials Figure S1: LC3 and p62 protein levels increase during starvation in wild type muscles, related to Figure 1. Immunoblotting analysis of wild-type EDL muscles subjected to 5 hours or 24 hours of starvation. Increases in LC3-II and p62 protein levels were detected. Actin protein levels were used as loading control. Figure S2: LC3 and p62 relative mRNA levels are modulated by leupeptin or colchicine treatment both in MCU^fl/fl^ and in skMCU^−/−^ mice, related to Figure 2. (a-d) Relative mRNA levels of LC3 and p62 in EDL muscles of skMCU^−/−^ or MCU^fl/fl^ mice treated or not with leupeptin (a-b) or colchicine (c-d) in fed conditions. ^∗^*p* < 0.05, *t*-test (two-tailed, unpaired) of 3 animals per condition. Data are presented as mean ± SE. (e-h) Relative mRNA levels of LC3 and p62 in EDL muscles of skMCU^−/−^ or MCU^fl/fl^ mice treated or not with leupeptin (e-f) or colchicine (g-h) in starving conditions. ^∗^*p* < 0.05, *t*-test (two-tailed, unpaired) of 3 animals per condition. Data are presented as mean ± SE.
