# Supplementary material for: Crosstalk between Mitochondrial Ca2+ Uptake and Autophagy in Skeletal Muscle
Source: Oxid Med Cell Longev. 2019 Sep 8;2019:1845321. doi: 10.1155/2019/1845321 (PMC6754932; doi:10.1155/2019/1845321)
Supplement: Supplementary Materials — Figure S1: LC3 and p62 protein levels increase during starvation in wild type muscles, related to Figure 1. Immunoblotting analysis of wild-type EDL muscles subjected to 5 hours or 24 hours of starvation. Increases in LC3-II and p62 protein levels were detected. Actin protein levels were used as loading control. Figure S2: LC3 and p62 relative mRNA levels are modulated by leupeptin or colchicine treatment both in MCUfl/fl and in skMCU−/− mice, related to Figure 2. (a-d) Relative mRNA levels of LC3 and p62 in EDL muscles of skMCU−/− or MCUfl/fl mice treated or not with leupeptin (a-b) or colchicine (c-d) in fed conditions. ∗p < 0.05, t-test (two-tailed, unpaired) of 3 animals per condition. Data are presented as mean ± SE. (e-h) Relative mRNA levels of LC3 and p62 in EDL muscles of skMCU−/− or MCUfl/fl mice treated or not with leupeptin (e-f) or colchicine (g-h) in starving conditions. ∗p < 0.05, t-test (two-tailed, unpaired) of 3 animals per condition. Data are presented as mean ± SE. [file 1845321.f1.pdf]

Supplementary Material

Crosstalk between mitochondrial Ca^{2+} uptake and autophagy in skeletal muscle

Gaia Gherardi, Giulia Di Marco, Rosario Rizzuto and Cristina Mammucari

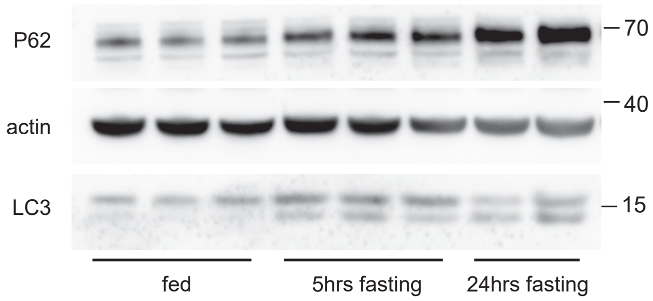

Fig S1. LC3 and P62 protein levels increase during starvation in wild type muscles, related to Figure 1. Immunoblotting analysis of wild type EDL muscles subjected to 5 hours or 24 hours of starvation. Increases in LC3-II and p62 protein levels were detected. Actin protein levels were used as loading control.

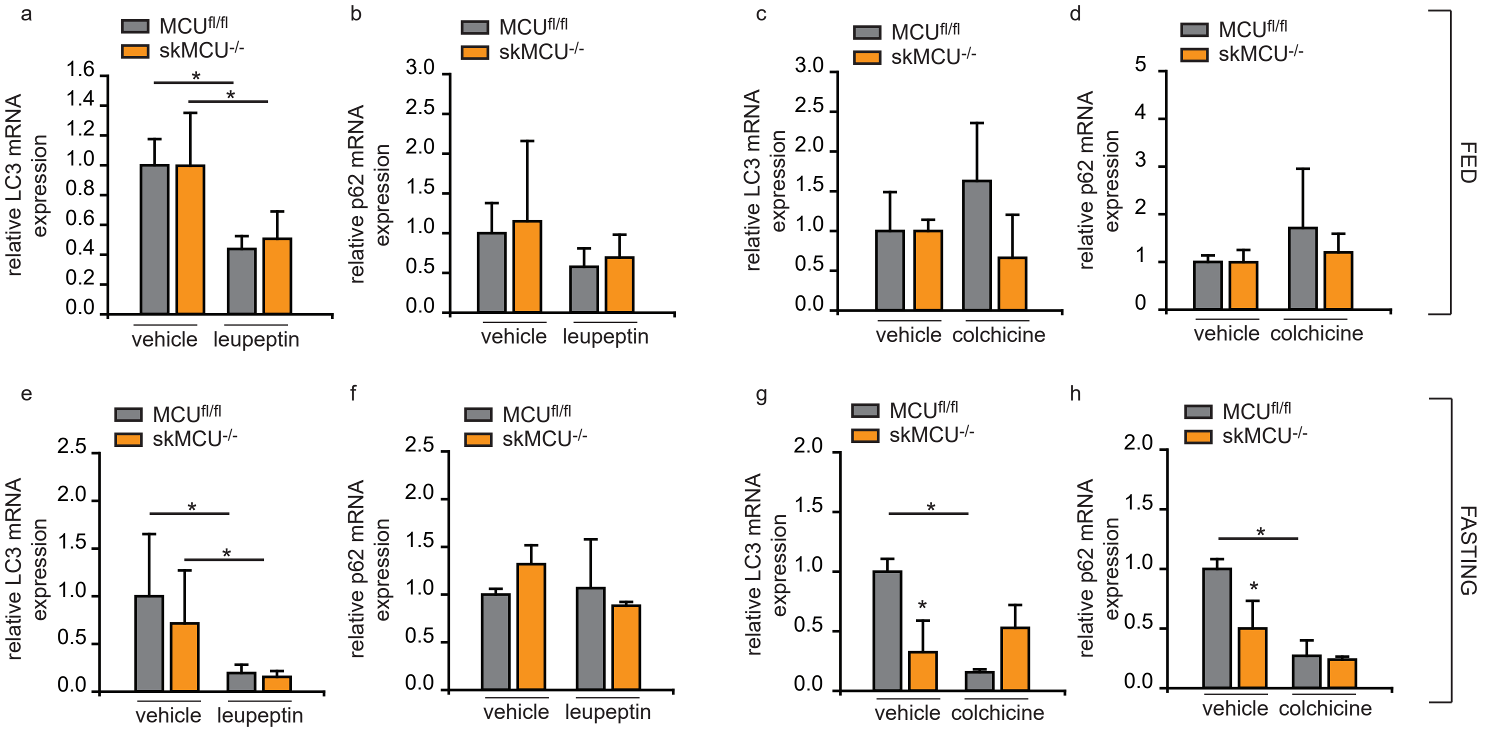

Fig S2. LC3 and p62 relative mRNA levels are modulated by leupeptin or colchicine treatment both in MCU$^{fl/fl}$ and skMCU$^{-/-}$ mice, related to Figure 2.

(a-d) Relative mRNA levels of LC3 and p62 in EDL muscles of skMCU$^{-/-}$ or MCU$^{fl/fl}$ mice treated or not with leupeptin (a-b) or colchicine (c-d) in fed conditions. *p <0.05, t- test (two-tailed, unpaired) of 3 animals per condition. Data are presented as mean ±SE.

(e-h) Relative mRNA levels of LC3 and p62 in EDL muscles of skMCU$^{-/-}$ or MCU$^{fl/fl}$ mice treated or not with leupeptin (e-f) or colchicine (g-h) in starving conditions. *p <0.05, t- test (two-tailed, unpaired) of 3 animals per condition. Data are presented as mean ±SE.
